# Best Practices and Joint Calling of the HumanExome BeadChip: The CHARGE Consortium

**DOI:** 10.1371/journal.pone.0068095

**Published:** 2013-07-12

**Authors:** Megan L. Grove, Bing Yu, Barbara J. Cochran, Talin Haritunians, Joshua C. Bis, Kent D. Taylor, Mark Hansen, Ingrid B. Borecki, L. Adrienne Cupples, Myriam Fornage, Vilmundur Gudnason, Tamara B. Harris, Sekar Kathiresan, Robert Kraaij, Lenore J. Launer, Daniel Levy, Yongmei Liu, Thomas Mosley, Gina M. Peloso, Bruce M. Psaty, Stephen S. Rich, Fernando Rivadeneira, David S. Siscovick, Albert V. Smith, Andre Uitterlinden, Cornelia M. van Duijn, James G. Wilson, Christopher J. O’Donnell, Jerome I. Rotter, Eric Boerwinkle

**Affiliations:** 1 School of Public Health, Human Genetics Center, The University of Texas Health Science Center at Houston, Houston, Texas, United States of America; 2 Medical Genetics Institute, Cedars-Sinai Medical Center, Los Angeles, California, United States of America; 3 Cardiovascular Health Research Unit, University of Washington, Seattle, Washington, United States of America; 4 Illumina, Inc., San Diego, California, United States of America; 5 Division of Statistical Genomics, Department of Genetics, Washington University School of Medicine, St. Louis, Missouri, United States of America; 6 Department of Biostatistics, Boston University School of Public Health, Boston, Massachusetts, United States of America; 7 Framingham Heart Study of the National, Heart, Lung, and Blood Institute, Framingham, Massachusetts, United States of America; 8 Institute of Molecular Medicine, Center for Human Genetics, The University of Texas Health Science Center at Houston, Houston, Texas, United States of America; 9 Icelandic Heart Association, Research Institute, Kopavogur, Iceland; 10 Faculty of Medicine, University of Iceland, Reykjavík, Iceland; 11 Laboratory of Population Science, National Institute on Aging, Bethesda, Maryland, United States of America; 12 Center for Human Genetic Research and Cardiovascular Research Center, Massachusetts General Hospital, Boston, Massachusetts, United States of America; 13 Harvard Medical School, Boston, Massachusetts, United States of America; 14 Broad Institute of Harvard and MIT, Cambridge, Massachusetts, United States of America; 15 Department of Internal Medicine, Erasmus University Medical Center, Rotterdam, The Netherlands; 16 Department of Epidemiology and Prevention, Wake Forest University School of Medicine, Winston-Salem, North Carolina, United States of America; 17 Department of Medicine, University of Mississippi Medical Center, Jackson, Mississippi, United States of America; 18 Department of Epidemiology, University of Washington, Seattle, Washington, United States of America; 19 Department of Medicine, University of Washington, Seattle, Washington, United States of America; 20 Department of Health Services, University of Washington, Seattle, Washington, United States of America; 21 Group Health Research Institute, Seattle, Washington, United States of America; 22 Center for Public Health Genomics, University of Virginia, Charlottesville, Virginia, United States of America; 23 ErasmusAGE and Department of Epidemiology, Erasmus University Medical Center, Rotterdam, The Netherlands; 24 Netherlands Consortium for Healthy Aging, Netherlands Genomics Initiative, Leiden, The Netherlands; 25 Department of Physiology and Biophysics, University of Mississippi Medical Center, Jackson, Mississippi, United States of America; 26 Cardiology Division, Massachusetts General Hospital, Boston, Massachusetts, United States of America; 27 Human Genome Sequencing Center, Baylor College of Medicine, Houston, Texas, United States of America; Institute of Cytology & Genetics SD RAS, Russian Federation

## Abstract

Genotyping arrays are a cost effective approach when typing previously-identified genetic polymorphisms in large numbers of samples. One limitation of genotyping arrays with rare variants (e.g., minor allele frequency [MAF] <0.01) is the difficulty that automated clustering algorithms have to accurately detect and assign genotype calls. Combining intensity data from large numbers of samples may increase the ability to accurately call the genotypes of rare variants. Approximately 62,000 ethnically diverse samples from eleven Cohorts for Heart and Aging Research in Genomic Epidemiology (CHARGE) Consortium cohorts were genotyped with the Illumina HumanExome BeadChip across seven genotyping centers. The raw data files for the samples were assembled into a single project for joint calling. To assess the quality of the joint calling, concordance of genotypes in a subset of individuals having both exome chip and exome sequence data was analyzed. After exclusion of low performing SNPs on the exome chip and non-overlap of SNPs derived from sequence data, genotypes of 185,119 variants (11,356 were monomorphic) were compared in 530 individuals that had whole exome sequence data. A total of 98,113,070 pairs of genotypes were tested and 99.77% were concordant, 0.14% had missing data, and 0.09% were discordant. We report that joint calling allows the ability to accurately genotype rare variation using array technology when large sample sizes are available and best practices are followed. The cluster file from this experiment is available at www.chargeconsortium.com/main/exomechip.

## Introduction

Exome- and whole-genome sequencing is becoming increasingly affordable and allows for detection and genotyping of rare variants in the human genome. Yet, genotyping arrays remain a cost-effective approach when investigating genetic polymorphisms previously identified in large populations. A limitation of using arrays to genotype rare variants is the difficulty that automated clustering algorithms have to accurately detect and assign accurate genotype calls [1], [2]. Large sample sizes increase the number of occurrences of rare variants and, therefore, should facilitate automated clustering and genotyping.

An array focused on rare and low frequency coding variation, hereafter referred to as the exome chip, has been developed by querying the exomes sequenced in ∼12,000 individuals and aggregating the variation that is seen in more than two individuals in more than two sequencing efforts (http://genome.sph.umich.edu/wiki/Exome_Chip_Design). Participating studies in the Cohorts for Heart and Aging Research in Genomic Epidemiology (CHARGE) Consortium [3] consented to have their Illumina Infinium HumanExome BeadChip intensity data analyzed collectively (n = 62,266) in order to increase the accuracy of rare variant genotype calls. The resulting cluster file (.egt) is publically available and we show that its use, along with best practices, increase genotype accuracy compared to other methods alone.

## Results

Genotypes were obtained for 238,876 successful variants in accordance with our best practices (96.4% SNP pass rate) which were converted to PLINK format [4] by cohort and combined into a single aggregate file for further analyses. Of the 62,266 samples genotyped, 1,380 (2.2%) had a GenCall quality score in the lower 10^th^ percentile of the distribution across all variants genotyped (p10GC) <0.38 or call rate <0.97 and were excluded from allele frequency calculations. Because founder effects and unique population structure have been previously observed in Icelandic samples [5], [6], the Age, Gene/Environment, Susceptibility-Reykjavik study was excluded from subsequent steps. Known duplicated samples, individuals without self-reported race, and the HapMap controls were also removed. After excluding duplicate variants (n = 811), the minor allele frequencies (MAF) for 238,065 successful SNPs and 56,407 samples by self-reported race are described in Table 1. There were 10,693 monomorphic SNPs (4.5%), and 78.6% of the variants on the exome chip have a MAF <0.005. Allele frequencies for each variant by race group are reported in the SNP information file (see Methods and Data Access sections). Ethnicity specific HapMap allele frequencies for the 96 controls (48 CEU and 48 YRI) and genotypes are also available.

**Table 1:** Exome chip minor allele frequency distribution by race.

MAF Interval	African Americans	Caucasians	Hispanics	Asians	All
	(n = 13,375) (%)	(n = 40,102) (%)	(n = 2,128) (%)	(n = 776) (%)	(n = 56,407) (%)
0	23.6	16.5	43.6	77.5	4.5
(0, 0.001]	36.8	58.1	22.3	3.9	58.8
(0.001, 0.005]	14.6	8.6	13.9	3.9	15.3
(0.005, 0.01]	4.4	2.3	3.7	1.6	4.3
(0.01, 0.05]	7.9	3.8	5.1	3.0	5.7
(0.05, 0.1]	2.7	1.7	1.7	1.4	1.8
(0.1, 0.2]	3.0	2.4	2.4	2.1	2.4
(0.2, 0.5]	7.1	6.7	7.3	6.6	7.3

The following samples were excluded: all AGES individuals, race unknown or not reported, known replicates, HapMap controls, individuals with p10GC <0.38, and individuals with call rate <0.97. Individuals with race designated as other were included in the overall MAF calculation, but data is not shown separately (n = 26). A total of 238,065 variants were used for calculating minor allele frequencies after excluding those that failed laboratory quality control (n = 8,994) and duplicates (n = 811).

To evaluate the performance of the rare variant calling approach (see Methods), we compared exome chip genotypes derived from three calling methods to available exome sequencing data in 530 ARIC individuals. First, exome chip genotypes were called with the Illumina issued cluster file HumanExome-12v1.egt (see Data Access section for file location) (Dataset I). Second, we used zCall (threshold set to 7) [7] to determine genotypes for the missing variant calls in Dataset I to create Dataset Z. Third, we used the CHARGE best practices (see Data Access) and joint calling approach described to ascertain exome chip genotypes (Dataset C). A total of 185,119 variants that were present in the exome sequence dataset and passed our best practices were compared using genotype concordance and uncertainty coefficient tests. Results are presented in Table 2. The uncertainty coefficients indicate that we can predict 86.4% of the information (entropy) in the exome sequence data when using the Illumina cluster file, 91.2% when using the zCall algorithm, and 93.4% when the CHARGE clustering method was utilized.

**Table 2:** Results of missing data, genotype discordance, uncertainty coefficients and frequencies of exome chip data ascertained by three calling methods and compared to exome sequence genotypes.

Exome Sequence	Exome Chip					Missing	Discordance	Uncertainty
Genotypes	Genotypes					(%)	(%)	Coefficient
**Dataset I**	**AA**	**AB**	**BB**	**XX**	**Total**			
**AA**	94,878,501	33,679	4,467	183,952	95,100,599			
**AB**	41,395	2,350,644	4,777	15,967	2,412,783			
**BB**	3,658	4,642	495,626	1,809	505,735			
**XX**	89,104	2,905	711	1,233	93,953			
**Total**	95,012,658	2,391,870	505,581	202,961	98,113,070	0.30	0.09	0.864
**Dataset Z**	**AA**	**AB**	**BB**	**XX**	**Total**			
**AA**	94,964,611	115,849	4,394	15,745	95,100,599			
**AB**	41,864	2,365,462	5,137	320	2,412,783			
**BB**	3,557	5,635	496,351	192	505,735			
**XX**	89,480	2,996	714	763	93,953			
**Total**	95,099,512	2,489,942	506,596	17,020	98,113,070	0.11	0.18	0.912
**Dataset C**	**AA**	**AB**	**BB**	**XX**	**Total**			
**AA**	95,023,653	33,442	4,430	39,074	95,100,599			
**AB**	41,850	2,363,664	3,969	3,300	2,412,783			
**BB**	3,606	4,646	496,897	586	505,735			
**XX**	89,391	2,930	706	926	93,953			
**Total**	95,158,500	2,404,682	506,002	43,886	98,113,070	0.14	0.09	0.934

A total of 185,119 variants were used for these analyses, excluding duplicated variants, short insertion/deletions, XY chromosome SNPs, Y chromosome SNPs, mitochondrial SNPs, sites not identified in the exome sequencing dataset, and failing SNPs as identified by the CHARGE best practices guidelines. Genotype classes are represented as AA = common variant homozygote, AB = heterozygote, BB = rare variant homozygote, and XX = missing data. Dataset I: exome chip genotypes called with Illumina cluster file. Dataset Z: zCall assigned genotypes to missing data in Dataset I. Dataset C: exome chip genotypes called with the CHARGE cluster file.

**Figure 1:**
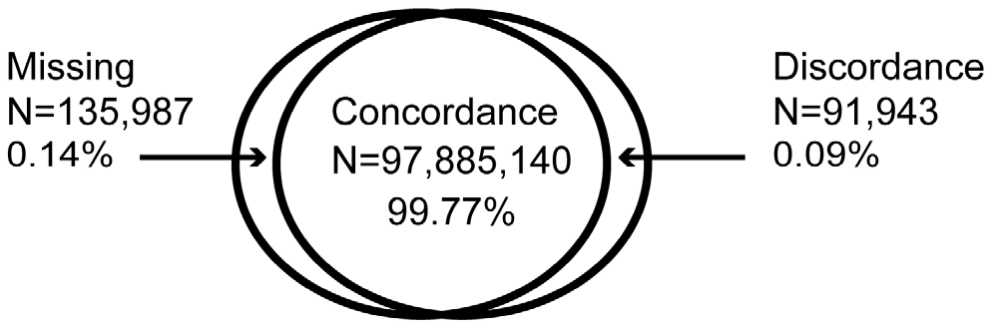
Results of CHARGE exome chip genotype calls compared to exome sequence data in 530 individuals.

These data demonstrate the importance of implementing stringent laboratory quality control measures in addition to the clustering algorithms and rare variant calling approaches tested. The complete list of 8,994 failing SNPs identified in the jointly called exome chip project are available for download on the CHARGE public website. Genotypes ascertained with the CHARGE jointly called exome chip cluster file (Dataset C) were 99.77% concordant with sequence data, 0.14% were missing in exome chip data, in the exome sequence data, or both, and 0.09% were discordant (Figure 1). Heterozygotes in Dataset C were most often misclassified when compared to the common allele homozygote, and mismatches were attributed equally to both sequencing and genotyping (Table 2).

We also tested the ability of the CHARGE exome chip cluster file to accurately assign genotypes in the three rarest variant bins: singletons (minor allele count = 1), doubletons (minor allele count = 2), and tripletons (minor allele count = 3). We observed high concordance between exome chip singletons (99.99%), doubletons (99.98%), and tripletons (99.97%) when compared to their respective sequence genotypes in the same 530 ARIC individuals previously described (data not shown). These results are consistent with the global concordance tests which suggest we are able to accurately call very rare variants.

## Discussion

The results presented here demonstrate that rare variants on the exome chip can be accurately called when using a large, combined cluster file and best practices described when compared to existing clustering algorithms and rare variant calling methods. The joint calling protocol, accompanying cluster file, list of poor performing variants on the chip, and annotation data are a valuable resource for the scientific community and will be of great utility to those having smaller sample sets where the calling of rare variants is problematic. All new projects will require user decisions based on their own cohort data and the metrics and best practices presented here should be updated accordingly.

## Materials and Methods

### Subjects

Data from 62,266 participants from the following eleven studies in the Cohorts for Heart and Aging Research in Genomic Epidemiology (CHARGE) Consortium [3] were included in this joint calling experiment and study descriptions were published previously: Age, Gene/Environment, Susceptibility-Reykjavik (AGES) Study [8], Atherosclerosis Risk in Communities (ARIC) Study [9], Cardiac Arrest Blood Study (CABS) [10], Cardiovascular Health Study (CHS) [11], [12], Coronary Artery Risk Development in Young Adults (CARDIA) [13], [14], Multi-Ethnic Study of Atherosclerosis (MESA) [15], Family Heart Study (FamHS) [16], Framingham Heart Study (FHS) [17], Health, Aging, and Body Composition (HABC) Study [18], Jackson Heart Study (JHS) [19], and the Rotterdam Study (RS) [20]–[23]. In addition, we genotyped 96 unrelated HapMap samples (48 CEU and 48 YRI) with each cohort and the list of sample IDs are available as a reference on the CHARGE exome chip public website.

### Ethics Statement

All subjects provided written and informed consent to participate in genetic studies, and all study sites received approval to conduct this research from their local respective Institutional Review Boards (IRB) as follows: „The National Bioethics Committee“ and „The Data Protection Authority“ (AGES); University of Mississippi Medical Center IRB (ARIC – Jackson Field Center), Wake Forest University Health Sciences IRB (ARIC – Forsyth County Field Center), University of Minnesota IRB (ARIC – Minnesota Field Center), and Johns Hopkins University (Bloomberg School of Public Health) IRB (ARIC – Washington County Field Center); University of Washington IRB (CABS); Wake Forest University Health Sciences IRB (CHS – Forsyth County Field Center), University of California, Davis IRB (CHS – Sacramento County Field Center), Johns Hopkins University (Bloomberg School of Public Health) IRB (CHS – Washington County Field Center), and University of Pittsburgh IRB (CHS – Pittsburgh Field Center); University of Alabama at Birmingham (CARDIA – Birmingham Field Center), Northwestern University IRB (CARDIA – Chicago Field Center), University of Minnesota IRB (CARDIA – Minneapolis Field Center), and Kaiser Permanente IRB (CARDIA – Oakland Field Center); Washington University IRB (FamHS); Boston University IRB (FHS); Wake Forest University Health Sciences IRB (HABC); University of Mississippi Medical Center IRB (JHS); Columbia University IRB (MESA – New York Field Center), Johns Hopkins University IRB (MESA – Baltimore Field Center), Northwestern University IRB (MESA – Chicago Field Center), University of California IRB (MESA – Los Angeles Field Center), University of Minnesota IRB (MESA – Twin Cities Field Center), Wake Forest University Health Sciences IRB (MESA – Winston-Salem Field Center) and the National Heart, Lung, and Blood Institute; Medisch Ethische Toetsings Commissie (METC) at the Erasmus Medical Center, and the Netherlands Ministry of Health, Welfare and Sport (VWS) (RS). Joint calling of the array data was approved by the Committee for the Protection of Human Subjects (CPHS) which serves as the IRB for the University of Texas Health Science Center at Houston.

### Genotyping

Study samples were processed on the HumanExome BeadChip v1.0 (Illumina, Inc., San Diego, CA) querying 247,870 variable sites described elsewhere (see Data Access) using standard protocols suggested by the manufacturer at the following seven genotyping centers: Broad Institute (JHS), Cedars-Sinai Medical Center (CHS, FamHS and MESA), Erasmus Medical Center (RS), Illumina Fast Track Services (FHS), University of Texas Health Science Center at Houston (AGES, ARIC and CARDIA), University of Washington (CABS), and Wake Forest University (HABC). Each center genotyped a common set of 96 HapMap samples to be utilized for quality control and determination of batch effects. The two channel raw data files (.idat) for all samples were transferred to a central location and assembled into a single project for joint calling. A summary of the samples genotyped within each cohort by race and gender is described in Table 3. The following variables were provided for each sample included in the project: study specific sample ID, cohort name, sample type (DNA or WGA), race (self-reported), gender, sample plate, sample well, chip barcode, chip position, replicate ID, father and mother IDs (if applicable).

**Table 3:** Sample sizes of cohorts participating in joint calling effort by gender and self-reported race.

Cohort	African Americans		Caucasians		Hispanics		Asians		Other		HapMaps		Replicates			Total
	M	F	M	F	M	F	M	F	M	F	M	F	M	F	U^1^	
AGES	0	0	1,305	1,767	0	0	0	0	0	0	24	24	6	9	0	3,135
ARIC	1,121	1,832	5,198	5,873	0	0	0	0	0	0	77	76	62	90	200	14,529
CABS	283	172	3,701	1,174	0	0	0	0	0	0	57	59	93	29	0	5,568
CHS	318	526	2,008	2,603	0	0	1	3	14	13	48	46	25	34	0	5,639
CARDIA	900	1,185	1,063	1,189	0	0	0	0	0	0	48	48	18	30	0	4,481
FamHS	213	409	933	1,191	0	0	0	0	0	0	14	23	1	0	0	2,784
FHS	0	0	3,702	4,475	0	0	0	0	0	0	75	69	47	76	0	8,444
HABC	515	680	930	839	0	0	0	0	0	0	48	47	7	5	0	3,071
JHS	1,063	1,795	0	0	0	0	0	0	0	0	64	64	0	0	0	2,986
MESA	1,129	1,464	1,282	1,397	978	1,151	387	386	0	0	44	44	51	39	0	8,352
RS	0	0	1,459	1,720	0	0	0	0	0	0	47	47	0	0	4	3,277
**Total**	**5,542**	**8,063**	**21,581**	**22,288**	**978**	**1,151**	**388**	**389**	**14**	**13**	**546**	**547**	**310**	**312**	**204**	**62,266**

1 Gender is unavailable for blinded replicates in the ARIC study and four RS samples.

### Clustering, Genotype Calling and Laboratory Quality Control

The Illumina GenomeStudio v2011.1 software was utilized with the GenTrain 2.0 clustering algorithm. Genomic DNA study samples and HapMap controls with call rates >99% (n = 55,142) were used to define genotype clusters with races combined and reruns excluded. The no-call threshold was set to 0.15 and we excluded female Y SNPs when calculating SNP statistics. The genotype quality score, representing the 10^th^ percentile of the distribution of GenCall scores across all SNPs genotyped (p10GC), was visually examined in a scatter plot across all samples (Index vs. p10GC). Samples with an empirically determined p10GC <0.38 were identified as outliers and flagged for exclusion. The SNP parameters “Expected Number of Clusters of Y SNPs” and “Expected Number of Clusters of mtSNPs” were set to 2. Following automated clustering, all variants meeting the criteria provided in Table 4 (n = 107,175) were visually inspected and manually clustered, if possible, by two independent laboratory technicians. AA and BB theta deviation cutoffs were determined empirically. Variants removed from the HumanExome BeadChip v1.1 (n = 4,969) and cautious sites, as defined by the exome chip design committee (n = 333) (ftp://share.sph.umich.edu/exomeChip/IlluminaDesigns/cautiousSites/cautiousSite.sorted.sites), were also inspected. Samples with a call rate between 0.95 and 0.99 that had been previously excluded were brought back in to the project and re-inspected based on the criteria listed in Table 4. This additional review was necessary as the CHARGE exome chip project contains samples from multiple DNA sources and ethnicities that were genotyped at several centers. SNPs exhibiting obvious batch effects were excluded. After joint calling, reproducibility and heritability statistics, SNP statistics and sample statistics were updated and the SNP-level quality control criteria described in Table 5 were implemented. SNPs with reproducibility (rep) errors >2, parent-parent-child (PPC) error >1, or parent-child (PC) error >1 were not excluded, but were flagged and reported back to the participating studies for further investigation. A list of the 8,994 excluded variants is provided on the CHARGE exome chip website as cluster positions for these sites are zeroed out in the.egt file (note: all SNP statistics for these sites will be converted to zero when the cluster file is imported into the Genome Studio project). A portion of excluded SNPs may be recoverable in projects with a homogenous population substructure, and we recommend clustering and reviewing the subset of variants with the user’s high quality samples. Table 6 describes the exome chip content and number of variants excluded by functional category (see Annotation). Importantly, the v1.0 cluster file should not be used for calling the Illumina v1.1 exome chip as the two versions were manufactured with different bead pools.

**Table 4:** Best practices criteria used to identify SNPs for visual inspection and manual reclustering.

Best Practices Criteria
All X, Y, XY and MT variants
Call frequency between 0.95 and 0.99
Cluster separation <0.4
AB frequency >0.6
AB R mean <0.2
Het excess >0.1
Het excess<−0.9
AA theta mean between 0.2 and 0.3
BB theta mean between 0.7 and 0.8
AB theta mean between 0.2 and 0.3
AB theta mean between 0.7 and 0.8
AA theta deviation >0.025
AB theta deviation ≥0.07
BB theta deviation >0.025
AB frequency = 0 and minor allele frequency >0
AA frequency = 1 and call rate <1
BB frequency = 1 and call rate <1
MAF <0.0001 and call rate ≠ 1
Rep error >2
PPC error >1
PC error >1
Variants removed from v1.1 exome chip
Cautious sites

AA: allele A homozygote; AB: heterozygote; BB: allele B homozygote; Het: heterozygote; MAF: minor allele frequency; MT: mitochondrial; PC: parent-child; PPC: parent-parent-child; R: normalized intensity; Rep, reproducibility.

**Table 5:** Exome chip SNP exclusion criteria.

Exclusion Criteria
Call frequency <0.95 (except Y chr)
Cluster separation <0.4
AB frequency >0.6
AB R mean <0.2
Het excess >0.1
Het excess<−0.9
AA theta mean >0.3
BB theta mean <0.7
AB theta mean <0.2 or >0.8
AA theta deviation >0.06
AB theta deviation ≥0.07
BB theta deviation >0.06
Obvious batch effects

AA: allele A homozygote; AB: heterozygote; BB: allele B homozygote; Het: heterozygote; R: normalized intensity.

**Table 6:** Exome chip content and CHARGE excluded variants by functional category.

Category^1^	Total Variants	Variants Excluded
exonic;stopgain	5,193	145
exonic;splicing;stopgain	90	1
exonic;stoploss	239	2
exonic;splicing;stoploss	5	0
splicing	2,263	60
exonic;splicing;synonymous	3,363	74
exonic;splicing	70	1
exonic;splicing;nonsynonymous	5,237	105
exonic;nonsynonymous	208,779	7,369
exonic;synonymous	6,415	281
UTR3	518	46
UTR5	77	6
ncRNA_splicing	1	1
ncRNA_exonic	111	8
ncRNA_UTR3	8	0
ncRNA_UTR5	1	0
intronic	5,762	254
ncRNA_intronic	447	23
downstream	187	19
upstream	181	7
upstream;downstream	8	0
intergenic	8,549	528
indel	137	10
mitochrondrial	226	54
no annotation	3	0
Total	247,870	8,994

1 dbNSFP was used for annotating variants [27] (see Methods).

### Exome Chip Performance

Genotypes derived from available exome sequencing of 540 ARIC participants were used as the comparison dataset to test the performance of the exome chip. We excluded 10 individuals from the sequencing dataset due to a high missing data rate <0.90, or non-overlap of individuals with existing exome chip data. Exome sequencing data is accessible via dbGaP as part of the National Heart Lung and Blood Institute (NHLBI) GO-ESP: Heart Cohorts Component of the Exome Sequencing Project (ARIC) (Study Accession: phs000398.v1.p1).

The following variants were excluded from the exome chip dataset as they were not available in the genotype data derived from exome sequencing results: replicate sites that were determined as triallelic or duplicates on opposite strands, short insertion/deletions, XY chromosome SNPs, Y chromosome SNPs, mitochondrial SNPs, or sites not identified in the exome sequencing dataset (n = 56,042). Poor performing variants identified by our best practices criteria were removed if not previously excluded (n = 6,709), thus a total of 185,119 variants were available for concordance analyses in 530 individuals.

Since concordance results are potentially high due to rare variation on the exome chip, we also calculated uncertainty coefficients [24] to determine the degree of association between each of the exome chip calling methods and exome sequence data. The uncertainty coefficient is a measure of association that is based on information entropy [25], or the uncertainty in a random variable, that is, a variable subject to chance variations. Uncertainty coefficients are useful when evaluating results obtained from clustering algorithms since genotype classification is usually random (all minor alleles are not classified as either AA or BB), thus the algorithm is not susceptible to rare variation bias in which the more common genotype could have been called by chance alone. See Press et al. (1992), pp. 758–762, for further clarification of the uncertainty coefficient metric [26].

### Annotation

Annotation of the v1.0 exome chip variants was performed with dbNSFP [27]. The dbNSFP v2.0 annotations are available on the CHARGE exome chip public website in the SNP information file. dbSNP rs information has been curated and a look up table with the associated Illumina SNP name is also available. The reason for inclusion of the variant on the exome chip by the design team is also provided in the SNP info file (ftp://share.sph.umich.edu/exomeChip/IlluminaDesigns/annotatedList.txt).

### Data Access

The following CHARGE supporting documents are located at chargeconsortium.com/main/exomechip: CHARGE_ExomeChip_Best_Practices.pdf, CHARGE_ExomeChip_v1.0_Cluster_File.egt (cluster file for v1.0 chip), CHARGE_ExomeChip_v1.0_Excluded_Variants.txt (list of 8,994 zeroed out variants in cluster file), CHARGE_ExomeChip_SNP_Info_File.tsv.txt and Read Me file includes Illumina annotation, dbNSFP annotation, dbSNP rs numbers, overlapping sites between the HumanExome BeadChip v1.0 and v1.1, reason for inclusion, and race specific allele frequencies for each variant, including HapMap controls. Sample identifiers (CHARGE_ExomeChip_HapMap96_Control_List.csv) and genotypes for the 96 unrelated HapMap controls (CHARGE_ExomeChip_HapMap96_Genotype_Data.csv) are also available.

The Illumina genotyping protocol (Infinium_Best_Practices_370-2009-010.pdf) and cluster file (HumanExome-12v1.egt) are available with a MyIllumina login at https://icom.illumina.com/. The exome chip content data sheet is publicly available at http://www.illumina.com/documents/products/datasheets/datasheet_humanexome_beadchips.pdf.

zCall is a rare variant caller for array-based genotyping provided by Goldstein et al. and available for download at github.com/jigold/zCall [7]. PLINK is a freely available analysis toolset at http://pngu.mgh.harvard.edu/purcell/plink/ [4].
